# Review of mathematical models of *Neisseria gonorrhoeae* vaccine impact: Implications for vaccine development

**DOI:** 10.1016/j.vaccine.2024.03.068

**Published:** 2024-03-30

**Authors:** Thilini N. Padeniya, Ben B. Hui, James G. Wood, David G. Regan, Kate L. Seib

**Affiliations:** a Institute for Glycomics, Griffith University, Gold Coast, Queensland, Australia; b The Kirby Institute, UNSW Sydney, New South Wales, Australia; c School of Population Health, UNSW Sydney, New South Wales, Australia

**Keywords:** Mathematical model, Vaccination, Gonorrhoea, Gonorrhea, Neisseria gonorrhoeae

## Abstract

An effective prophylactic vaccine for prevention of *Neisseria gonorrhoeae* infection would have a major impact on sexual and reproductive health worldwide. Interest in developing gonorrhoea vaccines is growing due to the reported high rates of *N. gonorrhoeae* infections globally, and the threat of antimicrobial resistance. Several gonorrhoea vaccine candidates are currently under evaluation and various mathematical models have been used to assess the potential population-level impact a gonorrhoea vaccine may have once available. Here we review key aspects of gonorrhoea vaccine mathematical modelling studies, including model structures, populations considered, and assumptions used as well as vaccine characteristics and implementation scenarios investigated. The predicted vaccine impact varied between studies, ranging from as little as ~17 % reduction in *N. gonorrhoeae* prevalence after 30 years up to 100 % reduction after 5 years. However, all studies predicted that even a partially effective gonorrhoea vaccine could have a substantial impact in reducing *N. gonorrhoeae* prevalence or incidence, particularly when high coverage is achieved within either important risk groups or the overall sexually active population. As expected, higher vaccine efficacy against acquisition of *N. gonorrhoeae* and longer duration of protection were linked to greater reductions in infections. A vaccine that alters onward transmission could also substantially reduce infections. Several gaps and research needs have been identified by researchers in the field and via this narrative literature review. For example, future modelling to inform gonorrhoea vaccine development and implementation should consider additional populations that are at high risk of *N. gonorrhoeae* infection, especially in low- and middle-income settings, as well as the impact of vaccination on the potential adverse sexual and reproductive health outcomes of infection. In addition, more detailed and robust epidemiological, biological, and behavioural data is needed to enable more accurate and robust modelling of gonorrhoea vaccine impact to inform future scientific and public health decision-making.

## 1. Introduction

*Neisseria gonorrhoeae* is a major public health concern, and the development of a gonorrhoea vaccine is considered a priority for improving sexual and reproductive health worldwide. There are an estimated 82 million cases of gonorrhoea worldwide each year [1] and the ongoing emergence and spread of antimicrobial resistant (AMR) *N. gonorrhoeae* strains raises concerns about maintaining effective treatment options [2]. Isolates with resistance to ceftriaxone, the last option for empirical first-line monotherapy, have been reported in several countries over the last decade [3–6]. A promising novel antibiotic treatment, zoliflodacin, is currently in phase 3 clinical trials (NCT03959527) for treatment of *N. gonorrhoeae* and, if proven to be effective, will provide another treatment option in the absence of a vaccine [7].

*N. gonorrhoeae* typically infects urogenital, rectal, or oropharyngeal mucosae and can result in a range of serious morbidities. In women, between 50 and 90 % of infections are asymptomatic, while in men, genital infection is typically characterised by symptomatic urethritis but may be asymptomatic in a proportion of patients [8–10]. If left untreated, *N. gonorrhoeae* infections in women can cause serious complications including pelvic inflammatory disease, ectopic pregnancy, spontaneous abortion, pre-term or low weight birth, stillbirth, neonatal blindness and infertility [11]. Complications of untreated infection in men include urogenital tract abscesses, orchitis, and prostatitis. Pharyngeal and anal infections are typically asymptomatic but can cause symptomatic pharyngitis and proctitis, respectively [12]. Gonorrhoea may also increase the risk of human immunodeficiency virus (HIV) acquisition and transmission [13,14].

The World Health Organization (WHO) Global Health Sector Strategy on sexually transmitted infections (STIs) has set a target for reducing gonorrhoea incidence by 90 % by 2030 and highlights the need for an effective vaccine in achieving this goal [15]. There are several gonorrhoea vaccine candidates currently in preclinical development [16,17] and there is increasing evidence that certain vaccines for the closely related organism *Neisseria meningitidis* may provide cross protection against *N. gonorrhoeae* [18–22]. Observational studies have detected a decrease in incidence of gonorrhoea following vaccination with the *N. meningitidis* serogroup B outer membrane vesicle (OMV)-based vaccines MeNZB [18] and 4CMenB [19–22], with a predicted vaccine efficacy of 30–40 % against *N. gonorrhoeae* infection. While a vaccine that completely prevents infection is ideal, this may not be achievable given the difficulties in developing a gonorrhoea-specific vaccine to date [23], and future vaccines may only provide partial protection against infection, and/or transmission. The WHO Preferred Product Characteristics (PPC) for gonococcal vaccines, which are intended to promote innovation and development of products for use in settings most relevant to the global unmet public health needs, include 50–70 % efficacy or greater, and at least 10–15 years duration of protection for vaccinating young adolescents without a booster but highlights that a vaccine with 3–5 years duration could still provide benefits for older age-groups and specific high-risk populations [11,16]. However, additional data from clinical trials and mathematical modelling will help refine the vaccine characteristics required to control *N. gonorrhoeae.*

The WHO STI vaccine roadmap [24] and the WHO PPC for gonococcal vaccines [11,16] have highlighted the importance of mathematical modelling to aid vaccine development. For example, transmission dynamic models can help define the vaccine characteristics needed to provide maximum health and economic impacts in different settings. They can also help identify optimal implementation strategies for different target populations. The aim of this narrative literature review is to describe the key aspects of gonorrhoea vaccine mathematical modelling studies conducted to date, including model structure, populations considered, assumptions used, and vaccine characteristics and implementation scenarios investigated. We also summarise the predicted impact of vaccines and vaccination scenarios considered in these studies and highlight remaining gaps and questions to be considered in future modelling studies to further inform vaccine development and implementation strategies.

## 2. Methods

**Fig. 1.**
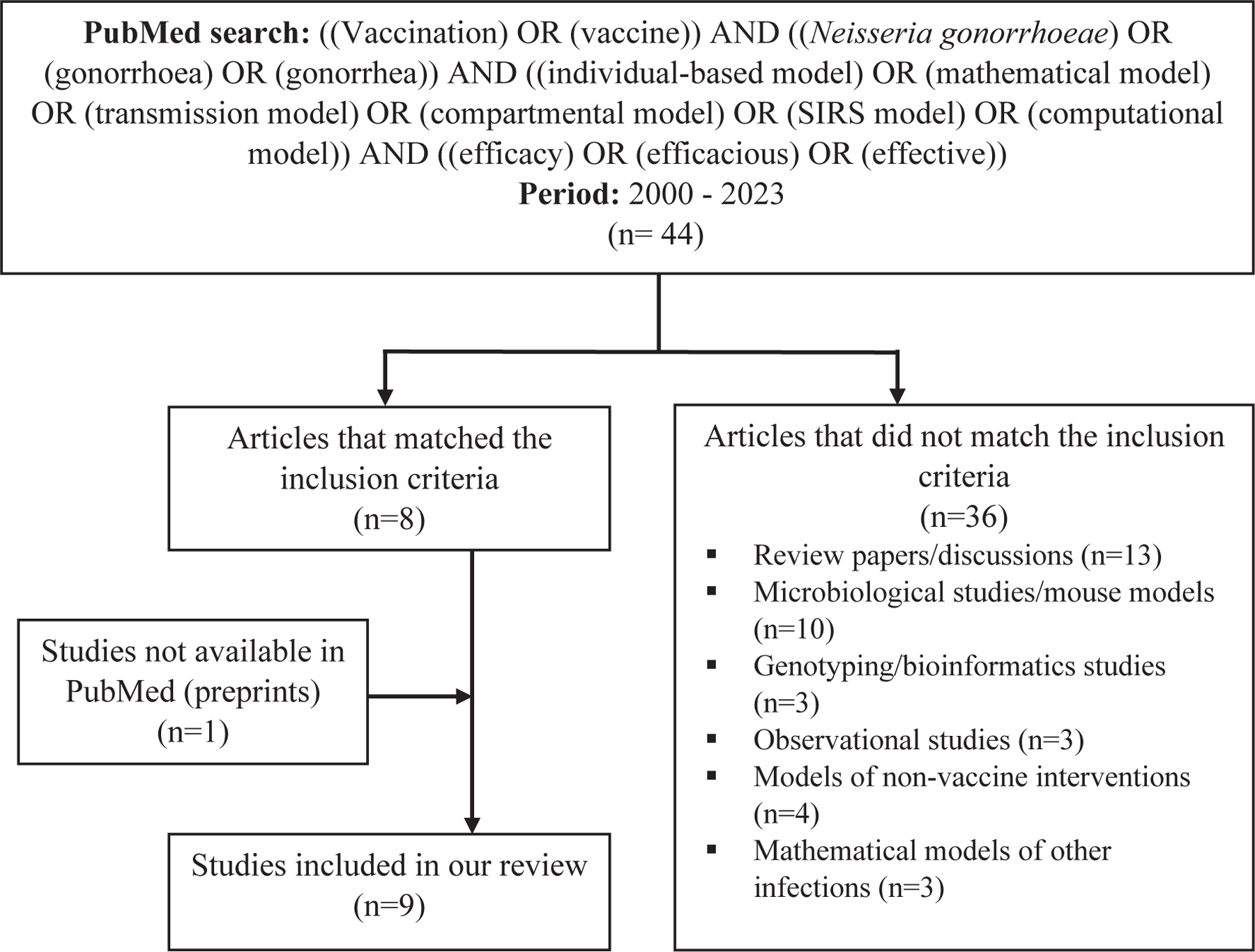
Process of identification of publications that describe dynamical models of *N. gonorrhoeae* transmission and assessed the impact of vaccination on *N. gonorrhoeae* prevalence/incidence for inclusion in this review.

We searched PubMed to identify peer-reviewed publications meeting the following inclusion criteria: dynamical models of *N. gonorrhoeae* transmission used to assess the potential impact of vaccination on *N. gonorrhoeae* prevalence/incidence, disease outcomes or antimicrobial resistance under different assumptions regarding vaccine efficacy. We used the following search terms: ‘((Vaccination) OR (vaccine)) AND ((*Neisseria gonorrhoeae*) OR (gonorrhoea) OR (gonorrhea)) AND ((individual-based model) OR (mathematical model) OR (transmission model) OR (compartmental model) OR (SIRS (Susceptible - Infectious - Recovered - Susceptible) model) OR (computational model)) AND ((efficacy) OR (efficacious) OR (effective))’ to find relevant literature published between January 2000 and August 2023 (Fig. 1). References cited in these publications and articles that cited the filtered publications were also considered to identify additional relevant publications. We extracted information from the identified studies that explained the key features and assumptions of each modelling study including target population, model calibration method, model type and method of modelling of *N. gonorrhoeae* transmission (Table 1), vaccine characteristics (Table 2), vaccine roll-out strategies (Table 3), predicted outcomes (Tables 4 and 5) and important/uncertain natural history parameter values assumed in reviewed models (Table S1).

## 3. Results

### 3.1. Modelling studies included in this review

Forty-four potentially relevant publications from 2000 to 2023 were identified via a PubMed search as described above. Of these, only eight mathematical modelling studies that focused on *N. gonorrhoeae* vaccines were identified as being of interest for this review. Additionally, one pre-print of a mathematical modelling study of gonococcal vaccination was identified. In total, nine studies are included in our review (see Fig. 1).

### 3.2. Characteristics of the models

The *N. gonorrhoeae* vaccine studies described in this review used deterministic compartmental [25–29], stochastic compartmental [30], or individual-based sexual network [31,32], or power-law sexual network [31–33] models. Table 1 summarises the key characteristics of these models, and Tables 2 and 3 define the vaccine characteristics and roll-out strategies considered, respectively. Table 5 provides an overview of each study in terms of the type of model, the setting and baseline *N. gonorrhoeae* prevalence/cases, vaccine characteristics and implementation strategies, and the predicted outcomes. The modelled outcomes differ between studies, with some considering reduction in prevalence and others the number of cases/diagnoses (Table 1). For simplicity, when discussing the models in general we refer to impact on *N. gonorrhoeae* “infection”, which may indicate either prevalence or the number of cases or diagnoses but when referring to individual models we use the specific outcome considered in that study.

### 3.3. Target population

Gonorrhoea incidence and prevalence varies between and within countries, with the greatest number of infections occurring in low- and middle-income countries (LMIC), and in key populations who are at higher risk of infection including: men who have sex with men (MSM); people in prisons; sex workers; transgender people; and people living with HIV [11]. The highest incidence of gonococcal infection typically occurs in 15 to 24-year-olds, with peak incidence in young adults (20–24 years), although incidence may also be high in older age-groups in populations at higher risk for infection [11]. Five modelling studies considered MSM populations with an age-range across models of 15–80 years and baseline *N. gonorrhoeae* prevalence of 3.4 % [29] or 12 % [32] or 98 *N. gonorrhoeae* diagnoses per year [33] or 20,000–35,000 *N. gonorrhoeae* cases per year [28,30]. Four studies considered heterosexual populations in the age-range 13–64 years across models, with a baseline prevalence of ~0.1 % [25], ~1–2 % [26,31], or ~3 % [27]. Modelled settings included the United Kingdom [25,28,30,33], the United States [26], Australia [32], the Netherlands [29], South Africa [27], and an unspecified generic setting [32]. All models considered an open population where people enter and leave the population at a specified rate. Out of all these models, two studies stratified the modelled population by age [25,27], while nine included stratification by sexual activity [25–33]. One model considered the anatomical site of infection (urethra/cervix, rectum, pharynx) and the different routes of transmission from infected to susceptible individuals depending on the anatomical site of infection in the infected individual and the type of sexual act (i.e., oral, anal, penile/vaginal, etc.) [32].

### 3.4. Model calibration

Model calibration is conducted to ensure that the behaviour of the pre-intervention model reflects the reported levels of *N. gonorrhoeae* infection in a specific setting (e.g., diagnoses, notifications, prevalence or incidence) before the introduction of the interventions being evaluated. In studies considering MSM populations, Whittles *et al.,* [28,30] varied natural history and behavioural parameters (e.g., per-partnership transmission probability, rate of recovery after treatment, probability that incident infection is symptomatic, rate of asymptomatic screening) in order to produce outcomes comparable to reported annual numbers of gonorrhoea tests and diagnoses and the proportion of diagnosed infections that are symptomatic. Heijne *et al.,* [29] varied the parameter that defines mixing between sexual activity classes, transmission probability per sex act and activity class-specific testing uptake, to match the reported activity class-specific percentage of *N. gonorrhoeae* diagnoses in Dutch MSM. In the anatomical-site-specific model developed by Hui *et al.,* [32], the transmission probability for each type of sexual contact was varied in order to calibrate the model to the reported anatomical-site-specific prevalence of urethral, anorectal, and oropharyngeal *N. gonorrhoeae*, while keeping other parameters fixed or sampled from predefined prior distributions.

In studies considering heterosexual populations, Craig *et al.,* [31] calibrated their model of heterosexual *N. gonorrhoeae* transmission, by varying the per-act transmission probability to produce the target prevalence (1.6–1.7 % based on prevalence data from different regions such as Europe and Africa [27]). Carey *et al.,* [26] identified 10,000 uncorrelated parameter sets, using Approximate Bayesian Computation, that produced baseline equilibrium prevalence matching the prevalence reported for the US (1.125 % and 0.75 % in females and males, respectively). All model parameters were allowed to vary except male-to-female per-act transmission probability, rate of shifting from high-to low-activity, and rate of people entering and leaving the population. In the study by Looker *et al.,* [25], the per-partnership transmission probability, rate of recovery, and sexual behaviour parameters were varied to calibrate the model to data on new gonorrhoea diagnoses among women and men in sexual and reproductive healthcare settings, adjusting for the number of diagnoses in MSM and considering the fact that all infections are not diagnosed. Padeniya *et al.,* [27] calibrated their model by adjusting the average partner acquisition rate for the entire population, the proportion of acts in which condoms are used in sexual partnerships, and the average number of sexual acts per week to reproduce reported age- and gender-specific *N. gonorrhoeae* prevalence in a setting in South Africa.

### 3.5. N. gonorrhoeae infection and transmission

*N. gonorrhoeae* is primarily transmitted via genital, oral and anal sexual contact, infecting cervical, urethral, pharyngeal and rectal mucosal surfaces. Transmission is highly efficient with a high proportion of people being infected after a single exposure [34]. Based on central estimates reported in the reviewed studies, in models considering transmission among MSM, the assumed probability of transmission across models varied between 0.3 and 0.5 per-act [29,32] or between 0.3 and 0.6 per-partnership [28,30]. In Whittles *et al.*’s power-law network model [33], three within-partnership transmission rates (rather than probabilities) per year were reported for different network types (fully connected, static, and dynamic) at 0.0018, 0.11, and 24. The individual-based model developed by Hui *et al.,* [32] also incorporated anatomical-site-specific transmission which varied between 0.2 and 0.9 per-act depending on the route of transmission (i.e., urethra to rectum, urethra to pharynx, etc.). In heterosexual models, the transmission probability was assumed to be higher from males to females than from females to males both per-act (0.2–1 and 0.1–0.5, respectively [26,27,31]) and per-partnership (~0.6 and ~0.4, respectively) [25]. *N. gonorrhoeae* transmission in the modelled populations is also influenced by the sexual behaviour assumptions used. Whittles *et al.,* [33], Hui *et al.,* [32] and Craig *et al.,* [31] modelled concurrent partnerships among people in addition to regular and casual (short-term) partnerships in their individual-based models. All other models considered different sexual activity groups defined based on assumed annual partner change rates. Heijne *et al.,* [29], Carey *et al.,* [26], and Looker *et al.,* [25] allowed individuals to change their sexual behaviour over the course of their sexually active lives. The models by Looker *et al.,* [25] and Padeniya *et al.,* [27] are stratified by age, gender, and sexual activity, and have varying partner acquisition rates for each age-group. The model by Looker *et al.,* [25] is unique in also considering importation of infection assumed to correspond to transmission to and from MSM who also have sex with women.

The incubation period (time from acquisition of infection to symptoms) and latent period (time from acquisition of infection to becoming infectious) for *N. gonorrhoeae* infection are not well known and are generally assumed to be 2 to 7 days but may be longer [35,36]. In the two retrospective studies of men, by Sherrard *et al.,* [35] and Schofield *et al.,* [36] the mean incubation period was reported to be 6.2 (standard error of mean ± 3.8) and 8.3 days, respectively, which was calculated using the records of dates of infection and the onset of symptoms. In the human challenge study by Schmidt *et al.,* [37], the incubation period was estimated to be in the range of 1–6 days. The reviewed modelling studies assumed similar incubation periods to those reported in these studies. Whittles *et al.,* [28,30,33] assumed that after acquisition of *N. gonorrhoeae*, individuals initially pass through a short incubation period (assumed to be 2 to 10 days when they are assumed to be infectious) after which they either develop symptoms or remain asymptomatically infected. Hui *et al.,* [32] assumed that upon acquiring infection, individuals enter a non-infectious exposed (or latent) state and remain there for 4 days before becoming infectious either symptomatically or asymptomatically. Other models did not consider an incubation or latent period. It is generally accepted that individuals who have recovered from infection do not acquire long-lasting immunity and may be reinfected soon after [38,39], hence all models reviewed here assumed that either there is no immunity after resolution of an infection [25,26,28–31,33], or that individuals are immune for 7 days post infection before becoming susceptible to infection again [27,32].

*N. gonorrhoeae* infection is estimated to be asymptomatic in 50–90 % of cases in women [8,40–42] and 10–90 % of cases in men [10,43,44]. In the modelling studies reviewed here, the proportions of infections that are asymptomatic are comparable to the published literature and varied between 60 % and 80 % for women and between 10 % and 50 % for men in heterosexual models [25–27,31], and between 30 % and 90 % in MSM models [28–30,32,33]. Additional studies are needed to provide more accurate data for this parameter for which there is currently considerable uncertainty, particularly for males. All models assumed equal transmission probabilities for asymptomatic and symptomatic infections, and Hui *et al.,* [32] and Padeniya *et al.,* [27] stated that this is due to the absence of robust data to support different values. However, in the individual-based model developed by Craig *et al.,* [31] the probability of transmitting infection was assumed to depend on the bacterial load of the infected partner, which is modelled using a mathematical relationship resulting in initial exponential bacterial growth followed by exponential decay [45].

*N. gonorrhoeae* has developed resistance to most of the antibiotics used to treat it [2,46], and Whittles *et al.,* [30] and Heijne *et al.,* [29] considered *N. gonorrhoeae* AMR in their models. Whittles *et al.,* introduced a resistant strain from outside of the model population and assumed that individuals acquire infection with either an antibiotic resistant or antibiotic sensitive strain [28,30]. Heijne *et al.,* assumed that *N. gonorrhoeae* resistance developed within the model population itself and defines four groups of strains: fully sensitive to antibiotics, intermediate sensitivity, reduced sensitivity and resistant [29].

### 3.6. Modelled vaccine characteristics and roll-out strategies

There are currently no vaccines available that provide specific and targeted protection against *N. gonorrhoeae* infection thus all models reviewed here considered hypothetical vaccines with differing efficacies, durations of protection (Table 2), implementation strategies and vaccination uptakes (Table 3). It should be noted that the predicted outcomes of vaccination will vary depending on how uptake is modelled, such as whether it involves recurring annual vaccination or a one-off vaccination. Regarding vaccine characteristics, the models considered the full range of possible values for efficacy (0–100 % across models) and a wide range for duration of protection (1–20 years across models). All studies considered vaccines that protected against infection, while some also considered vaccines that reduce onward transmission of infection [27,32], the duration of infection [29] and/or development of symptoms [27,31,32]. Hui *et al.,* [32] also investigated the impact of a vaccine with reduced efficacy for pharyngeal infection using their anatomical site-specific model, given that the effectiveness of antibiotic treatment against pharyngeal gonorrhoea is typically lower than for urethral and rectal infections [47,48], and therefore a vaccine’s efficacy may also differ by anatomical site of infection.

There are several complex and interrelated factors to be considered in regard to vaccination implementation strategies including geographical location, the specific epidemiological, health infrastructure, and programmatic factors for a given setting, and the target subpopulations to be offered vaccination. Five different vaccine roll-out strategies were investigated across the models reviewed here (Table 3). Ideally, vaccination will be delivered in a way that protects all those at risk of *N. gonorrhoeae* infection before the onset of risk, and thus most studies considered offering vaccination prior to sexual debut [25,26,28,30,31]. Craig *et al.,* [31] also considered gender-specific vaccination prior to sexual debut. Padeniya *et al.,* [27] assessed the impact of several age-specific roll-out strategies, including vaccinating a proportion of the entire modelled population or 15–24-year-olds only.

Several studies considered offering vaccination to a core group of highly sexually active people. Craig *et al.,* [31] considered offering vaccination to just ~5 % of the population with a partner change rate of ~20 per annum. Padeniya *et al.,* [27] assessed vaccination of a high-activity group having a partner change rate of ~5 per annum (~40 % of the population). Whittles *et al.,* [33] considered offering vaccination to a core group that contains 20 % of the modelled MSM population but did not specify partner change rates of the core group. Heijne *et al* [29] considered offering vaccination to MSM based on their sexual activity class (low, medium, high depending on sexual partner change rates); Whittles *et al.,* and Hui *et al.,* considered vaccination of MSM when they attend sexual health clinics for STI testing [28,30,32], on *N. gonorrhoeae* diagnosis [28,30], or those reporting a high number of sexual partners (>5 partners per year) [28]. Provision of booster vaccination was considered in several studies [25,29,32] and Looker *et al.,* [25] explored the impact of catchup vaccination offered to adolescents.

### 3.7. Model-predicted outcomes

The models reviewed here predict that roll-out of vaccines with different characteristics can reduce *N. gonorrhoeae* infections to varying degrees in different target populations, from as little as a 17 % reduction in *N. gonorrhoeae* infections after 30 years [29] up to a 100 % reduction after 5 years [32]. Table 5 gives an overview of the scenarios considered in each model, and in Table 4 we highlight scenarios that are consistent with the WHO PPC for gonococcal vaccines (i.e., at least 50–70 % efficacy and 10–15 years’ duration of protection (or 3–5 years for specific high-risk groups)) and show the duration of time needed to achieve the WHO Global Health Sector Strategy target of ~90 % reduction in *N. gonorrhoeae* infections [15].

Various mechanisms underlying vaccine efficacy were also considered. Hui *et al.,* [32] and Padeniya *et al.,* [27] predicted that for vaccines with < 100 % protective efficacy, the reduction in prevalence will be higher if the vaccine also reduces infectiousness of breakthrough cases and thereby onward transmission. For example, the reduction in prevalence is predicted to be twice as high for a vaccine that is 25 % efficacious against both acquisition and transmission of infection than for a vaccine that is 25 % efficacious against infection acquisition only, if 30 % of MSM get vaccinated per-clinic-visit [32]. The model by Heijne *et al.,* [29] predicts that a vaccine that reduces duration of infection is not as effective as a vaccine that reduces susceptibility or transmissibility. They explain that this may be due to the relatively high per-act transmission probability for *N. gonorrhoeae.* A vaccine should ideally protect against infection at all sites of infection, particularly in MSM where pharyngeal infection is proposed to be a key factor in sustaining high prevalence [49,50]. Hui *et al.,* [32] demonstrated that even if a vaccine completely protects against urethral and anorectal infection, *N. gonorrhoeae* prevalence is reduced by only 33 % at 5 years if there is no protection against pharyngeal infection, compared to elimination in the same time frame if the vaccine completely protects against infection at all anatomical sites. Models that considered vaccines that reduce symptoms of infection (which may result in a lower proportion of cases being diagnosed and treated) gave differing results, potentially due to differences in the sexual behavior of the populations and vaccine roll-out strategies considered. Craig *et al.,* [31] saw no difference in reduction in prevalence when considering vaccines of 50 % efficacy against infection that either did or did not affect symptoms. Hui *et al.,* [32], however, showed that if the vaccine supresses symptoms, reductions in prevalence will be less than when the vaccine reduces susceptibility only, while Padeniya *et al.,* [27] showed that *N. gonorrhoeae* prevalence is likely to increase if vaccination reduces susceptibility to infection by ≤ 25 %, but suppresses symptoms in vaccinated people. Vaccines with 1–20-years duration of protection were considered across the studies reviewed here, and two studies showed that booster vaccination could increase the percentage reduction in prevalence/incidence [25,32].

Whittles *et al.,* [30] and Heijne *et al.,* [29] considered antibiotic resistance in their models. In general, a vaccine with higher efficacy, duration of protection and/or coverage was necessary to achieve a similar reduction in *N. gonorrhoeae* cases in the presence versus absence of treatment failure, or to prevent development of antibiotic resistance. For example, Whittles *et al.,* [30] showed that a vaccine of > 52 % efficacy against infection and 6 years duration of protection, provided to all MSM attending sexual health clinics, would be necessary to achieve > 90 % reduction in *N. gonorrhoeae* cases after 10 years if treatment for the antibiotic resistant strain always fails. However, in the absence of treatment failure, the same reduction in cases could be achieved with a vaccine of 40–50 % efficacy against infection and 2–4 years duration of protection. Heijne *et al.,* [29] showed that a vaccine with 30 % efficacy could delay AMR development by several years, but to completely prevent the development of antibiotic resistance, a minimum efficacy of 90 % and uptake of 40 % is needed if vaccinating only high-activity MSM.

Five different vaccine roll-out strategies were investigated across the models reviewed here (Table 3). Craig *et al.,* [31] and Carey *et al.,* [26] considered vaccination prior to sexual debut in heterosexual populations with a baseline *N. gonorrhoeae* prevalence of ~1–2 %. Craig *et al.,* [31] showed that prevalence could be reduced by 30–75 % within 10 years by vaccinating 100 % of 13-year-olds with a vaccine of 40–100 % protective efficacy and 20-year duration of protection. The study by Carey *et al.,* [26] showed that prevalence could be reduced by ~50–75 % by vaccinating 50 % of 15-year-olds with a vaccine of 30 % protective efficacy, and 5–8 year duration of protection [26]. Carey *et al.,* [26] did not consider age or detailed risk stratification, which may account for the more substantial impact seen despite lower vaccine duration of protection and coverage compared to Craig *et al.,* [31]. In a setting with a lower prevalence of ~0.1 %, Looker *et al.,* [25] predicted a 6 % reduction in incident infections within 10 years with 85 % vaccination uptake in 14-year-olds (vaccine with 31 % protective efficacy, 3 year’s duration of protection). Padeniya *et al.,* [27] considered vaccination by age-group in a heterosexual population in a low to middle-income setting with ~3 % *N. gonorrhoeae* prevalence. They showed that prevalence can be reduced by ~50 % or ~80 % in 10 years if annual vaccination uptake is 40 % of 15–24-year-olds or 15–49 years, respectively (vaccine with 25 % protective efficacy, 5 year’s duration of protection), and that even at a lower vaccine uptake of 5 % of the entire unvaccinated population per year, *N. gonorrhoeae* prevalence could be reduced by 35–60 % within 10 years with a vaccine of 25–50 % protective efficacy and 5-year’s duration of protection.

Whittles *et al.,* compared different vaccine roll-out strategies targeting MSM in the UK and found that even a fully protective vaccine lasting 20 years would reduce *N. gonorrhoeae* cases by only ~30 % in 10 years when vaccination was provided before entry into the sexually active population. However, with vaccination on attendance for STI testing/treatment, 90 % reduction could be achieved with a vaccine that provides only 60 % protection for 4 years. In these scenarios, vaccination is provided to all MSM. Hui *et al.,* [32] showed that prevalence could be reduced by > 60 % in 5 years if 30 % of MSM are vaccinated per-clinic-visit with a vaccine having ≥ 50 % protective efficacy for 2 years. Heijne *et al.,* [32] predicted that a > 60 % reduction in MSM prevalence would take 30 years with 40 % vaccine uptake by high-activity MSM with a vaccine of ≥ 50 % protective efficacy lasting 2 years.

Several models showed that vaccinating a proportion of high-activity individuals within a core group is more efficient than vaccinating a similar proportion of the overall population. According to Whittles *et al.,* [33], a fully protective vaccine provided at random to 20 % of MSM can reduce *N. gonorrhoeae* diagnoses by ~30 % in one year, while vaccinating 20 % of individuals with the highest propensity to form partnerships can reduce diagnoses by ~40 %. According to Padeniya *et al.,* [27], targeting a proportion of high-activity individuals rather than the same proportion of the entire population requires ~3 times fewer vaccine doses to reduce the prevalence in the entire population by an equivalent amount. Craig *et al.,* [31] showed that vaccinating 75 % of the high-activity group (~5% of the population) will have a similar impact as vaccinating 50 % of the entire 13-year-old heterosexual population.

Only one study performed a cost-effectiveness analysis of *N. gonorrhoeae* vaccination. Whittles *et al.* [28] showed that the likelihood of a vaccine being cost-effective was highly sensitive to vaccine implementation strategy, price, efficacy and duration of protection. Vaccination of MSM according to risk (i.e., individuals diagnosed with *N. gonorrhoeae* in sexual health clinics, and those attending sexual health clinics for screening who report a high number of partners even if uninfected) was identified as the most cost-effective strategy for vaccines of moderate efficacy and/or duration of protection, although vaccination on diagnosis is predicted to be more cost-effective for highly protective and long-lasting vaccines. If vaccination is implemented according to risk, with a vaccine of 31 % efficacy (similar to that estimated for the MeNZB and 4CMenB vaccines) and 18 months duration of protection after two-doses and 36 months after an additional booster, then the vaccine would need to cost <£50 per dose to have > 50 % probability of being cost-effective over a 10-year time frame. At a vaccine cost of £18 per dose, vaccination according to risk with a vaccine as described above is predicted to avert ~110,200 cases, saving ~£8 million over 10 years. At a vaccine cost of £85 per dose, no strategy considered was found to be cost-effective.

## 4. Discussion

Given the challenges with existing antibiotic treatments [3,51,52] in the face of expanding antimicrobial resistance, gonococcal vaccines are considered the best option for the long-term control of *N. gonorrhoeae* infections. The availability of a gonorrhoea vaccine would have major public health and economic value [11]. The recently released WHO PPC for gonococcal vaccines [11] aims to support and guide vaccine development and ensure that when a gonorrhoea vaccine becomes available it will have the optimal public health value, particularly for LMICs where the burden of *N. gonorrhoeae* infection is highest. Nine gonorrhoea vaccine modelling studies (8 published and 1 pre-print) were included in this review and many insights have been gained from these studies, such as key vaccine characteristics, target populations and how gonococcal vaccinations could be implemented to meet public health goals.

Although the settings were different, the modelling studies have shown that vaccination for gonorrhoea could help reduce *N. gonorrhoeae* infections markedly, even with partial efficacy, moderate duration of protection and modest vaccine uptake. Several modelling studies included here considered vaccines having different characteristics [27,29,31,32]. As expected, the higher the protective efficacy/transmission suppression efficacy the greater the impact, however vaccines that reduce the duration of infection in vaccinated individuals with breakthrough infections are also able to reduce prevalence, but to a lesser degree than a vaccine that reduces susceptibility/transmissibility [27,29,32]. The impact of a vaccine that reduces symptoms is less clear, with the effect on prevalence being different depending on the setting and the level of vaccine protection modelled [27,32]. Another consideration is the duration of protection and the number of doses that will be required to provide strong and lasting immunity. Program effectiveness increases with the duration of protection provided by a vaccine. However, delivery of booster vaccinations to extend the period of protection, while likely to be less efficient, can also be effective in reducing infections [25,32]. The impact of vaccination is also highly dependent on the target populations and vaccine roll-out strategies, and the models reviewed here considered heterosexual [25–27,31] or MSM [28–30,32,33] populations, with vaccination prior to sexual debut [25,26,28,30,31], annually [27,29], targeting high-activity groups or on attendance at a sexual health clinic [27–33].

Several studies showed that vaccinating a high-activity core group is highly effective in reducing *N. gonorrhoeae* infections and is typically more efficient that other strategies, requiring a lower cumulative number of vaccinations to achieve a similar outcome [27,31,33]. Identifying and accessing high-activity populations for vaccination may be challenging in some settings, however the approach of offering vaccination to individuals presenting for STI screening [28,30,32] is potentially a viable and pragmatic option and did results in substantial reductions in modelling studies that considered this strategy.

There are several limitations of existing studies, in terms of the settings and scenarios considered, as well as the epidemiological, biological, and behavioural data available for model calibration. The models described to date focus on a limited range of settings, with most considering heterosexual or MSM populations in high-income countries (HIC). Only one study considers a LMIC setting [27]. The WHO PPC for gonococcal vaccines highlights the importance of vaccinating key and vulnerable populations, such as MSM, sex workers, transgender people, people living with HIV, incarcerated people, and ethnic minorities/Indigenous populations [11,16] and focus should shift to considering these populations. Strengthening *N. gonorrhoeae* epidemiological data worldwide, including age- and gender-specific prevalence and incidence will be pivotal to accurately estimate the potential impact of vaccination in different populations and settings. Given the ongoing emergence and spread of AMR, and that a key aim for gonococcal vaccines is to reduce the impact of gonococcal AMR [11,16], improved AMR surveillance is also essential, particularly in LMICs which are typically underrepresented in countries reporting to the WHO’s Gonococcal Antimicrobial Surveillance Programme (GASP) [1]. Two models reviewed here considered AMR [29,30] but the other studies did not factor in increasing *N. gonorrhoeae* infections over the course of the model that may arise due to AMR, and this may result in overestimation of vaccine impact. Similarly, more robust data is needed for sexual behaviour in different populations (e.g., sex worker client rates and condom use), how sexual behaviours change over time, mixing between populations (e.g., mixing between different sexual networks), and key factors that drive transmission (e.g., duration of untreated infection, proportion of infections that are symptomatic, relative infectiousness of symptomatic/asymptomatic infection) to parameterise and calibrate models more accurately. Three studies considered movement between activity groups over time [25,26,29], and only one study considered mixing between heterosexual and MSM populations [25]. The possible impact of the availability of vaccination on sexual behaviour is not considered in any of the studies reviewed here. If the introduction of a gonococcal vaccination was to result in compensatory behavioural changes such as reduced condom-use, the reductions in *N. gonorrhoea*e infections would likely be overestimated in these studies. However, the widespread rollout of human papillomavirus vaccination has not been shown to have had a noticeable effect on sexual behaviour among sexually active young adults in the US, Canada, and African settings [53–56].

The current gaps in our understanding of various aspects of *N. gonorrhoeae* transmission dynamics and the sequelae of infection limit the level of detail and accuracy of model-derived predictions of vaccine impact. The substantial variations in the values for the per-act and per-partnership transmission probabilities across the reviewed studies, as outlined in Section (v) of the Results, shows the uncertainty around these parameter values. There is also a particularly large gap in available evidence regarding anatomical site-specific parameters (transmission probabilities, differential vaccine efficacy) and only one MSM study reviewed here [32] considered anatomical site-specific infection and vaccine efficacy. This highlights the need for well-designed empirical studies aimed at identifying more reliable and robust values for these parameters, although we note that studies of this nature, e.g., transmission studies in couples, are particularly challenging. Additionally, there is a need for more robust data in regard to the relationship between infectious dose, bacterial load during infection and risks of transmission and symptomatic infection. While associations between bacterial load and transmissibility have been reported [57], bacterial load does not always correlate with symptoms or the severity of infection [58,59] and the link between symptoms and transmission is unclear. Thus, all studies reviewed here assumed that both asymptomatic and symptomatic infections are equally transmissible. Craig *et al.,* [31] assumed that the probability of transmitting infection depends on the gonococcal load of the infected partner, but do not link bacterial load with the presence of symptoms. While model predictions for hypothetical downstream interventions may be associated with considerable uncertainty, robust model calibration to data that includes at least partial observations on the effect of these interventions can provide some confidence that model predictions lie within plausible ranges. Therefore, despite the challenges of imperfect data to parameterise models, modelling still provides important information regarding these public health issues.

Another key limitation of the current studies is that they only focus the number of *N. gonorrhoeae* infections and did not consider the potential adverse sexual and reproductive outcomes of gonococcal infection, such as pelvic inflammatory disease, adverse pregnancy outcomes neonatal conjunctivitis, infertility, disseminated infection, and impact on HIV acquisition and transmission [11,16]. Better estimates of the quality-adjusted life years (QALYs) or disability-adjusted life years (DALYs) lost due to gonococcal infections are required as all of the sequelae of *N. gonorrhoeae* infection also have economic and psychosocial consequences that need to be considered. Only one model reviewed here included a cost-effectiveness evaluation of vaccination [28], and as the protective characteristics of potential gonorrhoea vaccines become clearer, more thorough assessment of disease burden, costs, and vaccine cost-effectiveness will be needed. Since there is a need for a gonococcal vaccine in both HIC and LMIC settings, the vaccines need to be cost-effective and affordable. Thus, modelling studies assessing the cost-effectiveness of vaccination while considering the impact of long-term sequelae and increasing AMR are important to better understand vaccine impact in different settings. The WHO PPCs should be informed by cost-effectiveness studies and indicate price ranges for vaccines with different protective properties that may be acceptable in terms of cost in HIC and LMIC settings.

In the absence of a naturally acquired protective immune response following *N. gonorrhoeae* infection and the lack of a gonorrhoea-specific vaccine, the vaccine characteristics needed to provide protection are poorly understood. Observational and case-control studies have shown that *N. meningitidis* serogroup B vaccines MeNZB [18] and 4CMenB [19–22], may provide cross protection against *N. gonorrhoeae*, with a predicted efficacy in the range 30–40 %. Several clinical trials are now underway with 4CMenB [60] (clinicaltrials.gov NCT04350138, NCT04415424) and a gonorrhoea-specific vaccine (clinicaltrials.gov NCT05630859) that will hopefully generate key information regarding vaccine efficacy, as well as vaccine impact on symptoms and anatomical-site specific infection. Furthermore, since some of these studies are in MSM populations at high risk of STIs, the impact of previous *N. gonorrhoeae* infection or concurrent infection with other STI on vaccine efficacy may be revealed. There is evidence to suggest that people who have had prior gonococcal infection have antibodies to the protein Rmp that block the ability of other antibodies to kill *N. gonorrhoeae* [61]. If this is the case, then vaccination prior to sexual debut would be a high priority. However, prior gonococcal infection might prime the adaptive immune system, that is then boosted by vaccination. Interestingly, MeNZB was associated with lower effectiveness against *N. gonorrhoeae* when there was a coinfection with *Chlamydia trachomatis* [18].

As outlined above, future work is needed to fill the knowledge and data gaps in the field that will facilitate additional gonococcal vaccine modelling studies to accurately assess the potential impact of vaccination in LMIC settings and key target groups. Ideally, future modelling should incorporate more detailed and accurate data on *N. gonorrhoeae* incidence, prevalence, AMR, infection dynamics, and the role of coinfection with other STI. Similarly, more complex population structures should be modelled with respect to age, gender, and sexual behaviour and activity. To achieve a more complete understanding of vaccine impact, a range of the most important *N. gonorrhoeae* infection sequelae (e.g., pelvic inflammatory diseases, infertility, adverse pregnancy outcomes, impact on HIV transmission) should be included such that the full health and economic burden associated with infection can be estimated. To address some of these issues, existing models may be able to be modified by updating current parameters to better match epidemiological and behavioural patterns in different settings as better data become available. However, heterosexual models incorporating anatomical site-specific infection are needed to provide a better understanding of the potential impact vaccination on extragenital infections in a heterosexual population. New models may also be needed to accurately assess the impact of vaccination offered to different key populations. For example, high population mobility contributes to the high levels of STIs observed in remote Indigenous communities of Australia [62], and a new model that incorporates population movement between communities would enable a more accurate evaluation of potential vaccine impact in these communities [62].

While all mathematical modelling studies have their limitations, the studies reviewed here have provided valuable insights into the potential impact of vaccination and can inform gonococcal vaccine development and implementation. Gonococcal vaccine development has been reinvigorated in the past few years with more vaccines currently in clinical trials than in the past 30 years. As the vaccine development pipeline progresses, more relevant vaccine characteristics can be incorporated into models, along with more realistic vaccine roll-out strategies guided by how specific vaccine characteristics may address the needs of specific settings, with respect to issues such as target populations and available resources. Ongoing modelling efforts will substantially progress our understanding of the future impact of a gonococcal vaccine and help reduce AMR and improve sexual and reproductive health worldwide.

## Supplementary Material

Table S1

Appendix A. Supplementary data

Supplementary data to this article can be found online at https://doi.org/10.1016/j.vaccine.2024.03.068.

## Figures and Tables

**Table 1:** Characteristics of the *N. gonorrhoeae* vaccine models reviewed.

Characteristic	Number of Studies	Reference
*Model type:*		
Deterministic compartmental	5	[25–29]
Stochastic compartmental	1	[30]
Individual-based sexual network^†^	3	[31–33]
*Population Stratification:*		
By sexual activity	9	[25–33]
By anatomical site	1	[32]
By age-group	2	[25,27]
*Modelled population:*		
Heterosexual	4	[25–27,31]
Men who have sex with men (MSM)	5	[28–30,32,33]
*Setting:*		
Australia	1	[32]
UK	4	[25,28,30,33]
Netherlands	1	[29]
USA	1	[26]
South Africa	1	[27]
Unspecified	1	[31]*
*Main modelled outcomes:*		
Prevalence	5	[26,27,29,31,32]
Number of *N. gonorrhoeae* cases/diagnoses	3	[28,30,33]
Percentage reduction in incident infections	1	[25]
Prevalence of antibiotic resistant strains	2	[29,30]
Cost-effectiveness	1	[28]

* Used sexual behaviour data from various settings such as Netherlands and US to parameterise the model.

† This includes both individual-based and power-law sexual network models.

**Table 2:** Characteristics of *N. gonorrhoeae* vaccines investigated in the models reviewed.

Vaccine characteristics/efficacies	Definition	Reference
Efficacy in protecting against infection*	The percentage reduction in the risk of acquisition of infection among vaccinated individuals	[25–33]
Efficacy in reducing transmission*	The percentage reduction in the risk of transmission of infection by vaccinated individuals with breakthrough infection	[27,29,32]
Efficacy in reducing symptoms	The degree to which the vaccine reduces the development of symptoms in vaccinated individuals with breakthrough infection	[27,31,32]
Efficacy in reducing duration of infection	A partially protective vaccine reduces the duration of infection	[29]

* Some studies considered per-partnership efficacy [25,27,28,30] and others per-act efficacy [32], while in others this is not mentioned [26,29,31,33]. For a given per-partnership efficacy, the equivalent value for per-act efficacy is typically higher.

**Table 3:** Vaccine implementation strategies evaluated in the reviewed modelling studies.

Vaccine roll-out	Definition	Reference
Vaccination before entry to sexually active population	Provide vaccination to adolescents before they become sexually active (13–15 years old)	[25,26,28,30,31]
Vaccination on testing/treatment	Provide vaccination when individuals present at a sexual health clinic for routine STI testing/treatment regardless of *N. gonorrhoeae* infection status	[28,30,32]
Vaccination on diagnosis	Provide vaccination to individuals diagnosed with *N. gonorrhoeae* on presentation at sexual health clinics, both through seeking care for symptomatic infection and through attending for screening (testing for asymptomatic infection)	[28,30]
Vaccination according to risk	Provide vaccination to individuals diagnosed with *N. gonorrhoeae* in sexual health clinics, and those attending sexual health clinics for screening who report high-risk sexual behavior even if they test negative	[28]
Vaccinating specific age/activity groups	Provide vaccination to individuals belonging to a certain age/activity group annually	[27,29]
Booster vaccination	Provide revaccination after an initial vaccination course. E.g., vaccinated individuals receive booster vaccinations every 3 years on average to offset the effect of waning vaccine-conferred protection	[25,28,32]

**Table 4:** Efficacy and implementation characteristics of scenarios that led to 90% reductions in *N. gonorrhoeae* infections.

Context	Roll-out strategy	Efficacy against infection/transmission (%)	Duration of protection (years)	Time to achieve ~90 % reduction (years)*
Heterosexual setting (1.6–1.7 % prevalence) Craig *et al.,* [31]	100 % of 13-year-olds	60	20	18
Heterosexuals (US setting, 1–2 % prevalence) Carey *et al.,* [26]	50 % of 15-year-olds	50	5	10
Heterosexuals (similar to South African setting, 2.8 % prevalence) Padeniya *et al.,* [27]	10 % of unvaccinated people annually	50	10	10
Heterosexuals (UK setting, ~0.1 % prevalence) Looker *et al.,* [25]	Did not achieve 90 % reduction			
MSM (UK setting, (21,900 cases) Whittles *et al.,* [30]	All MSM attending sexual health clinics	60	4	10 (AMR is modelled)
MSM (UK setting, (~34,000 cases) Whittles *et al.,* [28]	33 % of MSM on attendance at a sexual health clinic	78	1.5 after two-doses, 3 after re-vaccination	10
MSM (Australian setting, (12 % prevalence) Hui *et al.,* [32]	60 % of MSM presenting for STI screening	75	2	2
MSM (Dutch setting, (3.4 % prevalence) Heijne *et al.,* [29]	40 % of the high-activity MSM susceptible to infection	70	2	30 (AMR is modelled)
MSM (UK setting, 98 diagnoses) Whittles *et al.,* [33]	Not reported			

**Table 5:** Summary of the main vaccination scenarios assessed, and predictions found from gonorrhoea vaccine modelling studies.

Article	Model type	Setting and Baseline prevalence/incidence	Model population stratification	Main vaccination scenario and uptake considered	Vaccine efficacy	Duration of protection (Year)	Reduction in Prevalence/incidence	Notes/other outcomes
Craig *et al.* (2015) [31]	Individual-based model	Heterosexual population (no specific setting) 1.6–1.7 % prevalence	Considered short- and long-term partnerships with durations of partnership 14 days and 8 yrs, respectively. Percentage in the core group is 5 % and they may have concurrent partnerships with average duration of 21 days.	100 % of 13-yr-olds	20 %-100 % against transmission	2.5–5	~0–40 % in 10 yrs.	Vaccinating 75 % of high-activity core group (5 % of population) achieved a similar population-level outcome as vaccinating 50 % of all 13-yr-olds.
	
						7.5–10	~70–80 % in 10 yrs.	
			
						20	~20–80 % in 10 yrs.	
					
				50 % of 13-yr-olds	100 %	20	~50 % in 10 yrs.
					
				13-yr-old females or 13-yr-old males	100 %	20	~50 % in 10 yrs.

Whittles *et al.,* (2019) [33]	Dynamic power-law sexual network model	MSM in the UK98 diagnoses	There are high- and low-degree individuals. Considered short-term, long-term, and concurrent partnerships but have not provided the values.	20 % of individuals at random	100 % against infection	Unspecified	30 % in 1 yr.	Vaccination had a greater impact than 20 % increase in condom-use, or 20 % increase in sexual health screening.
	
				20 % of core group	100 %	Unspecified	40 % in 1 yr.

Whittles *et al.,* (2020) [30]	Stochastic compartmental model	MSM in England 21,900 cases (95 % CI: 18,900–25,200)	Low-risk group: 85 % of the population with an average partner change rate of 0.6 per yr. High-risk group: 15 % of the population with an average partner change rate of 15.6 per yr.	Uptake: 50–100 % Before entry into the population	50–100 % against infection	7–20	10–40 % in 10 yrs.	In scenarios modelled, an antimicrobial resistant strain emerges in 2020, for which antibiotic treatment always fails.
	
				On diagnosis of gonorrhoea	50–100 %	7–20	50–100 % in 10 yrs.	
					
				On attendance at sexual health clinic	50–100 %	7–20	90–100 % in 10 yrs.

Whittles *et al.,* (2022) [28]	Deterministic compartmental model	MSM in England~34,000 cases	Same as above.	Uptake: 33 % (95 % CI: 32.7–33.3 %) Before entry into the population	40 % against infection	4	~6% in 10 yrs.	Vaccination according to risk is most cost-effective for vaccines of moderate efficacy and/or duration, while vaccination on diagnosis is most cost-effective for highly protective and long-lasting vaccines. They also considered 1 booster dose.
				On diagnosis			~30 % in 10 yrs.	
				Vaccination according to risk			~65 % in 10 yrs.	
				On attendance at sexual health clinic			~65 % in 10 yrs.	

Hui *et al.,* (2021) [32]	Anatomical-site-specific individual-based model	MSM in urban Australia 12 % prevalence (IQR:11–14 %)	Percentage with, only regular partnerships is 33 %, only casual partnerships is 26 %, both regular and casual is 41 %. Casual partner acquisition rate per 6 months, 51 % having 1–9 partners and 49 % having 10 + partners. The average duration of, casual partnerships is around 1.4 days, and regular partnership is 4yrs.	MSM attendance at sexual health clinic for regular testing Per-clinic-visit vaccination uptake: 30 %	0–100 % against infection and transmission	2	20–100 % in 5 yrs 40–100 % in 5 yrs if a vaccine booster is given every 3 yrs.	Outcomes indicate that vaccines need to be effective against pharyngeal infection for Ng prevalence to be substantially reduced, with elimination unlikely even if the vaccine efficacy at the urethra and anorectum is ~100 %.
	
					50 % symptom suppression and ≥ 50 % against infection and transmission	2	70–100 % in 5 yrs. NB- if efficacy against infection and transmission is *<* 50 %, prevalence increases at 5 yrs.

Carey *et al.,* (2022) [26]	Deterministic compartmental model	Heterosexuals in the US aged 15–24 yrs. 1.125 % women/0.75 % men prevalence	High-activity proportion: 15 %. Contact rate: ~290 per yr. Low-activity proportion: 85 %. Contact rate: ~3 per yr.	Portion prior to sexual debut 20 % 50 %	30–70 % against infection	2–8	9–70 % in 10 yrs. 22–100 % in 10 yrs.	Based on sensitivity analysis, the most important parameters for the variability of vaccine impact are the proportion of contacts reserved for assortative mixing and proportion of the population in the high-activity group.
		group. 2.25 % women/1.5 % men		20 % 50 %			4–34 % in 10 yrs. 11–98 % in 10 yrs.	

Looker *et al.,* (2023) [25]	Deterministic compartmental model	Heterosexuals in England aged 13–64 yrs. 0.08 % women/0.05 % men prevalence	Four activity groups (1, 2, 3,4) with mean number of 0, 1, ~2, and ~7 partners per year. Six age-groups (1, 2…6): 13yrs, 14yrs, 15–16yrs, 17–18yrs,19–24yrs and 25–64yrs. The proportion of population in activity group 1 is relatively higher in young age-groups (1 and 2) than the age-group 5 and 6 (~90 % vs 20 %). The proportion in activity groups 1 and 2 is comparatively higher than the proportion in activity groups 3 and 4	75–95 % of 14-yr-olds	20–50 % against infection	6	6–16 % of infections averted in 10 yrs.	Catch-up vaccinations and boosters could further reduce infections.

Heijne *et al.,* (preprint, 2020) [29]	Deterministic compartmental model	MSM in the Netherlands 3.4 % prevalence (95 % CI: 3.2–3.8 %)	Partners per year in, low-activity: 7, medium-activity: 13, high-activity: 36, Percentage of people in each of the above activity classes are, 72 %, 26 % and 2 %, respectively.	High-activity MSM susceptible to gonorrhoea Uptake: 20–80 %	30 % against infection	2 510	10–60 % in 30 yrs. 30–80 % in 30 yrs. 40–90 % in 30 yrs.	Results were similar if the vaccine reduces transmissibility, rather than susceptibility. A vaccine that reduces the duration of infection is less effective in reducing prevalence and delaying the time to develop antibiotic resistance.
	
				Uptake: 40 %	30–90 % against infection	2 510	30–100 % in 30 yrs. 50–100 % in 30 yrs. 60–100 % in 30 yrs.

Padeniya *et al.,* (2023) [27]	Deterministic compartmental model	Heterosexuals aged 15–49 yrs. (setting similar to South Africa) 2.8 % prevalence (IQR:2.6–3.0 %)	Stratified by low- and high-activity with average partner change rate of ~1 and ~5 per yr, respectively. Percentage population in high and low-activity groups is 35 % and 65 % with equal proportions of people in each age-group.	Uptake: 5–40 % Overall population	25–100 % against infection	2–10	−100 % in 10 yrs.	If the vaccine is efficacious against infection acquisition and onward transmission prevalence reductions are even greater. If the vaccine suppresses symptoms, reductions in prevalence will be less than when the vaccine reduces susceptibility only. However, if the vaccine reduces both susceptibility and transmissibility, a noticeable reduction in prevalence can be seen even if the vaccine suppresses symptoms.
				15–24-yr-olds			8–100 % in 10 yrs.	
				15–19-yr-olds			3–90 % in 10 yrs.	

MSM, men who have sex with men; Ng, *Neisseria gonorrhoeae*; core group, highly sexually active group; CI, confidence interval; IQR, interquartile range.

## Data Availability

No data was used for the research described in the article.
