# Supplementary material for: Review of mathematical models of Neisseria gonorrhoeae vaccine impact: Implications for vaccine development
Source: Vaccine. Author manuscript; Available in PMC 2025 Jul 13. (PMC12256113; doi:10.1016/j.vaccine.2024.03.068)
Supplement: Table S1 [file NIHMS2091668-supplement-Table_S1.pdf]

Review of mathematical models of *Neisseria gonorrhoeae* vaccine impact: Implications for vaccine development

Thilini N. Padeniya, Ben B. Hui, James G. Wood, David G. Regan, Kate L. Seib

Table S1: Values of some important/uncertain natural history parameters used in the reviewed modelling studies. The values listed here are the central estimates reported in the reviewed studies.

Parameter	Reviewed modelling Study	Values used in the models reviewed	Inferred through model fitting/assumption
**Per-act transmission probability**	Craig *et al.,* [1]	1 (male-females), 0.5 (female-male) at peak gonococcal load	Inferred through model fitting
	Padeniya *et al.,* [2]	0.28 (male-female), 0.15 (female-male)	Calibration to the age-specific data was aimed at identifying optimal parameter choices for the three key sexual behaviour parameters as discussed in Section IV of the Results. In addition to that, some natural history parameters, including gender specific per-act transmission probabilities have been varied to allow for some flexibility around model-predicted prevalence.
	Carey *et al.,* [3]	0.7 (male-female) ~0.4 (female-male)	The male-female transmission probability is fixed here (haven't mentioned where this value is obtained from), but the female-male is varied. In this study, the female-male transmission probability = contagiousness of women compared to men × male-female per-act transmission probability. They have varied the parameter contagiousness of women compared to men.
	Heijne *et al.,* [4]	0.37 (MSM)	Inferred through model fitting
**Per-partnership transmission probability**	Whittles *et al.*, [5]	0.3 (MSM)	Inferred through model fitting
	Whittles *et al.*, [6]	0.56 (MSM)	Inferred through model fitting

	Looker *et al.,* [7]	0.58 (male-female), 0.38 (female-male)	Inferred through model fitting
**Within-partnership rate of transmission per year**	Whittles *et al.*, [8]	Fully connected network: 0.0018<br>Static network: 0.11<br>Dynamic network: 24 (MSM)	Inferred through model fitting
**Anatomical-site-specific per-act transmission probability**	Hui *et al.,* [9]	0.867 (urethra-anorectum)<br>0.233 (anorectum-urethra)<br>0.886 (urethra-oropharynx)<br>0.157 (oropharynx-urethra)<br>0.354 (anorectum-oropharynx)<br>0.719 (oropharynx-anorectum)<br>0.292 (oropharynx to oropharynx)	Inferred through model fitting
**Duration of infection**	Whittles *et al.*, [5]	119 days	Inferred through model fitting
	Whittles *et al.*, [6]	174 days	Inferred through model fitting
	Whittles *et al.*, [8]	Fully connected network: 223 days<br>Static network: 167 days<br>Dynamic network: 166 days	Inferred through model fitting
	Looker *et al.,*	126 days (males),<br>187 days (females)	Inferred through model fitting
	Craig *et al.,*	137 days (males), 228 days (females)	Not reported
	Padeniya *et al.,*	180 days (average duration for both males and females)	Assumption based on the published data [10-12]
	Heijne *et al.,*	137 days	Assumption based on the published data [13-17]
	Hui *et al.,*	Urethral: 185 days<br>Anorectal: 360 days<br>Oropharyngeal: 84 days	Assumption based on the published data [11, 18-20]
	Carey *et al.,*	~60 days for males,<br>~90 days for females	Inferred through model fitting

References

1. Craig, A.P., et al., *The potential impact of vaccination on the prevalence of gonorrhea.* Vaccine, 2015. **33**(36): p. 4520-5 DOI: https://doi.org/10.1016/j.vaccine.2015.07.015.
2. Padeniya, T.N., et al., *The potential impact of a vaccine on Neisseria gonorrhoeae prevalence among heterosexuals living in a high prevalence setting.* Vaccine, 2023 DOI: http://doi.org/10.1016/j.vaccine.2023.07.048.
3. Carey, K.A., L.M. Newman, and I.H. Spicknall, *Estimating the population level impact of a gonococcal vaccine candidate: Predictions from a simple mathematical model.* Vaccine, 2022 DOI: https://doi.org/10.1016/j.vaccine.2022.10.031.
4. Heijne, J.C., et al., *The impact of vaccination on Neisseria gonorrhoeae antimicrobial resistance and prevalence in men who have sex with men: a mathematical modelling study.* medRxiv, 2020: p. 2020.09.14.20192062 DOI: https://doi.org/10.1101/2020.09.14.20192062.
5. Whittles, L.K., X. Didelot, and P.J. White, *Public health impact and cost-effectiveness of gonorrhoea vaccination: an integrated transmission-dynamic health-economic modelling analysis.* The Lancet Infectious Diseases, 2022 DOI: http://doi.org/10.1016/S1473-3099(21)00744-1.
6. Whittles, L.K., P.J. White, and X. Didelot, *Assessment of the potential of vaccination to combat antibiotic resistance in gonorrhea: a modeling analysis to determine preferred product characteristics.* Clinical Infectious Diseases, 2020. **71**(8): p. 1912-1919 DOI: https://doi.org/10.1093/cid/ciz1241.
7. Looker, K.J., et al., *The potential public health impact of adolescent 4CMenB vaccination on Neisseria gonorrhoeae infection in England: a modelling study.* BMC Public Health, 2023. **23**(1): p. 1 DOI: https://doi.org/10.1186/s12889-022-14670-z.
8. Whittles, L.K., P.J. White, and X. Didelot, *A dynamic power-law sexual network model of gonorrhoea outbreaks.* PLOS Computational Biology, 2019. **15**(3): p. e1006748 DOI: https://doi.org/10.1371/journal.pcbi.1006748.
9. Hui, B.B., et al., *A Gonococcal Vaccine Has the Potential to Rapidly Reduce the Incidence of Neisseria gonorrhoeae Infection Among Urban Men Who Have Sex With Men.* The Journal of Infectious Diseases, 2021 DOI: https://doi.org/10.1093/infdis/jiab581.
10. Hui, B.B., et al., *The potential impact of new generation molecular point-of-care tests on gonorrhoea and chlamydia in a setting of high endemic prevalence.* Sex Health, 2013. **10**(4): p. 348-56 DOI: http://doi.org/10.1071/SH13026.
11. Garnett, G.P., et al., *The transmission dynamics of gonorrhoea: modelling the reported behaviour of infected patients from Newark, New Jersey.* Philos Trans R Soc Lond B Biol Sci, 1999. **354**(1384): p. 787-97 DOI: http://doi.org/10.1098/rstb.1999.0431.
12. *World Health Organization. Prevalence and incidence: Chlamydia trachomatis, Neisseria gonorrhoeae, syphilis and Trichomonas vaginalis. Methods and results used by WHO to generate 2005 estimates.* 2011.
13. Xiridou, M., et al., *Cost-Effectiveness of Dual Antimicrobial Therapy for Gonococcal Infections Among Men Who Have Sex With Men in the Netherlands.* Sex Transm Dis, 2016. **43**(9): p. 542-8 DOI: http://doi.org/10.1097/olq.0000000000000480.
14. Tuite, A.R., et al., *Impact of Rapid Susceptibility Testing and Antibiotic Selection Strategy on the Emergence and Spread of Antibiotic Resistance in Gonorrhea.* J Infect Dis, 2017. **216**(9): p. 1141-1149 DOI: http://doi.org/10.1093/infdis/jix450.

15. Holmes, K.K., et al., *Sexually Transmitted Diseases.* 4th ed., New York: McGraw-Hill, 2008.
16. Kretzschmar, M., Y.T.H.P. van Duynhoven, and A.J. Severijnen, *Modeling prevention strategies for gonorrhea and chlamydia using stochastic network simulations.* American Journal of Epidemiology, 1996. **144**(3): p. 306-317 DOI: http://doi.org/10.1093/oxfordjournals.aje.a008926.
17. Barbee, L., et al., *O02.4 Incidence and duration of pharyngeal and rectal gonorrhea and chlamydia among high-risk men who have sex with men (MSM).* Sexually Transmitted Infections, 2019. **95**(Suppl 1): p. A40-A40 DOI: http://doi.org/10.1136/sextrans-2019-sti.113.
18. Johnson, L.F., L. Alkema, and R.E. Dorrington, *A Bayesian approach to uncertainty analysis of sexually transmitted infection models.* Sex Transm Infect, 2010. **86**(3): p. 169-74 DOI: http://doi.org/10.1136/sti.2009.037341.
19. Brunham, R.C., et al., *Gonococcal infection and human fertility in sub-Saharan Africa.* Proceedings of the Royal Society of London. Series B: Biological Sciences, 1991. **246**(1316): p. 173-177 DOI: http://doi.org/10.1098/rspb.1991.0141.
20. Fairley, C.K., et al., *Is it time to move to nucleic acid amplification tests screening for pharyngeal and rectal gonorrhoea in men who have sex with men to improve gonorrhoea control?* Sexual health, 2011. **8**(1): p. 9-11 DOI: http://doi.org/10.1071/SH10134.
